# Monitoring of rheumatoid arthritis: a patient survey on disease insight and possible added value of an innovative inflammation monitoring device

**DOI:** 10.1007/s00296-021-05026-8

**Published:** 2021-10-21

**Authors:** Ria Wolkorte, Lieke Heesink, Michelle M. A. Kip, Hendrik Koffijberg, Monique Tabak, Christiane Grünloh

**Affiliations:** 1 Health Technology and Services Research Department, Technical Medical Centre, University of Twente, Enschede, The Netherlands; 2 Biomedical Signals and Systems Group, Faculty of Electrical Engineering, Mathematics and Computer Science, University of Twente, Enschede, The Netherlands; 3 eHealth Group, Roessingh Research and Development, Enschede, The Netherlands

**Keywords:** Rheumatoid arthritis, Disease monitoring, Patient perspective, Shared decision making, Inflammation imaging, Patient education

## Abstract

To enable patients with rheumatoid arthritis (RA) and their healthcare professionals to choose the optimal treatment, it is crucial to accurately assess the current state of inflammatory activity. The objectives of this study were to (1) investigate the perspective of RA patients on their insight into the current status of their disease, and to (2) investigate the patients’ perspective on the possible added value of a monitoring device based on optical spectral transmission—called the HandScan—that measures the location and severity of joint inflammation. A survey was distributed online among patients with RA in the Netherlands. Four-hundred and eight patients with RA completed the survey. Of these, 298 (73%) felt they have sufficient insight into their current disease status. Most respondents perceived either a large (*n* = 242; 59%) or small (*n* = 148; 36%) added value of the HandScan in their monitoring process, mostly because the device provides additional knowledge on the presence of inflammation. This perceived added value was higher for respondents experienced with the device (*n* = 46; *p* = .04). Respondents preferred monitoring with the device on every (*n* = 192; 47%) or most (*n* = 171; 42%) visits to the outpatient clinic, or even more often than on every visit (*n* = 17; 4%). Monitoring RA using an optical spectral transmission device is seen by patients as a possibly valuable addition to the monitoring process of inflammatory activity during visits to an outpatient clinic. Their main reason was that the device can increase insight into their current disease status. More insight may support patients in discussing treatment options with their rheumatologist.

**Supplementary Information:**

The online version contains supplementary material available at 10.1007/s00296-021-05026-8.

## Introduction

Rheumatoid arthritis (RA) is a chronic condition that can have a severe impact on a person’s quality of life [1]. The main symptom is inflammation of the joints which may result in swelling, pain, stiffness and joint destruction [1, 2]. Early diagnosis and optimal treatment are imperative to improve long-term outcomes and minimize permanent joint damage [3].

Patient-centered care is considered very important in the treatment of patients with RA [4, 5]. A key aspect of patient-centered care is that patients are involved in the decision-making process for treatment [4, 6], which is associated with better adherence and treatment outcomes [6, 7]. The degree of involvement of patients and their role can vary and is related to several factors, including the severity of complaints and the complexity of the decision [8]. For patients to be involved in decision making, they need to have insight into all relevant factors, including their current disease status, treatment options, and the (dis)advantages of these options. Among patients with RA, there is a high need for information [9].

A patient's current RA status is commonly monitored through clinical examination by a trained rheumatologist, blood tests, and a set of imaging techniques for inflammation including MRI and ultrasound [10]. Furthermore, a disease activity score such as the DAS28 is calculated, which is based on a count of 28 swollen and tender joints, levels of inflammatory markers in the blood, and a VAS score on disease activity by the patient[11, 12]. As it is known that imaging techniques more accurately detect inflammation than clinical examinations, both the EULAR and ACR have recommended that imaging techniques are part of the monitoring process [10, 13, 14]. The frequency of imaging is decided by the rheumatologist.

**Fig. 1:**
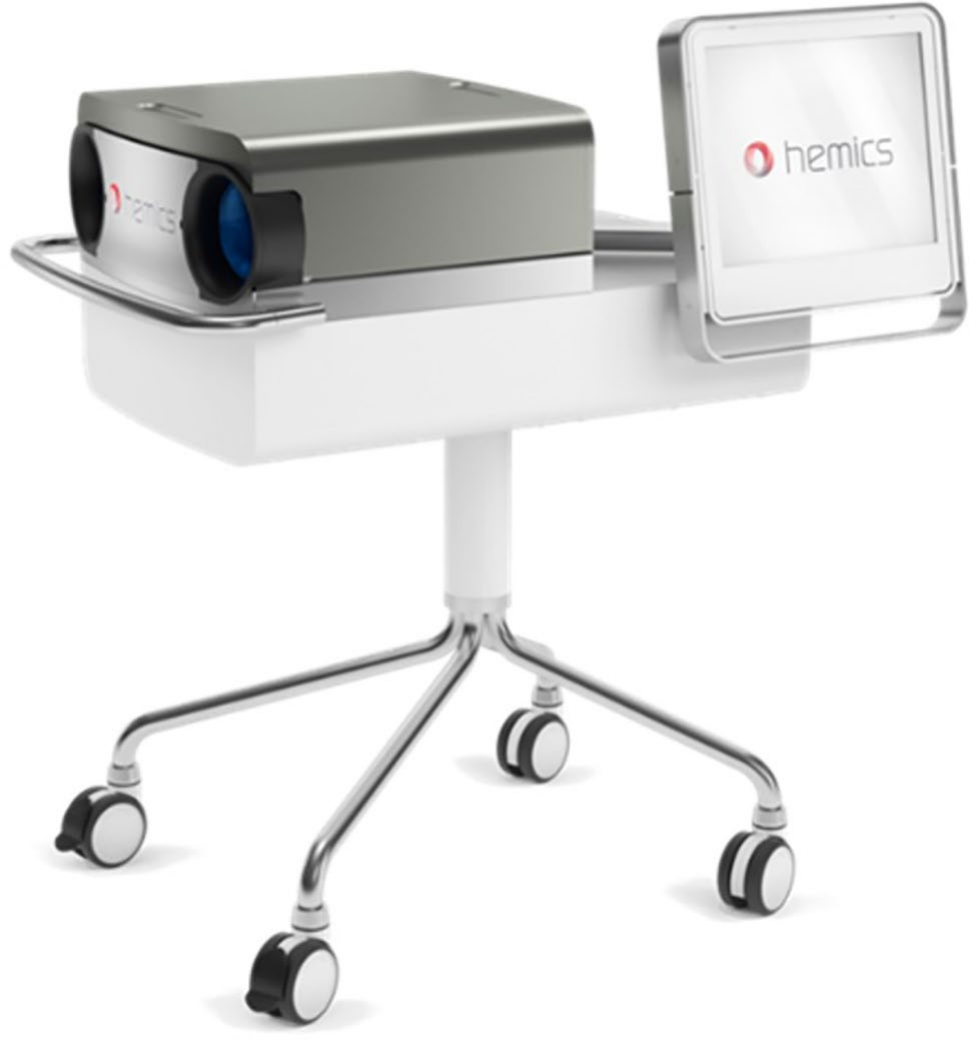
HandScan. The HandScan (Demcon Hemics, The Netherlands) is a new imaging device for the detection of inflammation. Patients place both hands in the device simultaneously, resting on a plate. Red/near-infrared light illuminates the hand and the amount of light that passes through the hand is recorded by a camera. The more blood is present, the less light is passed through. The total procedure takes approximately 5–10 min and can be executed by any healthcare professional after a short training [15]. The HandScan produces a value for the level of inflammation for each separate joint and a combined score for the entire hand. The inflammation is also shown visually with a scale from green to red superimposed on the joints of the hand and wrists. This may help to understand the current activity of a patient’s arthritis as well as to localize inflammatory activity. Previous research showed that the HandScan is a sensitive and specific test with good validity [15, 16]. This makes it interesting to examine the HandScan as a potential addition to or (partial) replacement of current measures for inflammatory activity such as blood tests, DAS28, MRI, and ultrasound

A device based on optical spectral transmission—the HandScan—has recently been developed for detecting inflammation [15]. Optical spectral transmission technology might be a valuable addition to or (partial) replacement of current measures for inflammatory activity such as the DAS28, blood tests, MRI and ultrasound [16]: it is an objective measure and the test can be performed by anyone after a short training. For more information on the HandScan, see Fig. 1.

The first aim of this study was to investigate patients’ opinions about their insight into their current disease status. The second aim was to investigate patients’ opinions on the potential added value of the HandScan to monitor inflammatory activity as part of secondary care.

## Method

### Design

An online survey was conducted from June to August 2020.

### Participants

Patients with RA, 16 years or older, were recruited regardless of any existing experience with the HandScan. Recruitment of a convenience sample took place online by sharing the survey among members and followers of national and local patient associations, via websites, and social media. One of the hospitals that use the HandScan in daily practice sent the link to the survey by email to their patients with RA.

Patients received information on the investigators, study purpose, procedure, approximate duration of the survey, data handling and privacy. They could contact the researchers with additional questions. They were required to provide informed consent before starting the survey. Five vouchers of 10 euro were randomly awarded among patients. The vouchers were provided as an appreciation for the effort on the part of the participants. Participants interested in receiving a voucher could enter a raffle to be eligible.

The study has been approved by the ethics committee of the faculty of Behavioural, Management and Social Sciences of the University of Twente (req. nr. 200865).

### Materials

The survey was developed with the input of experts on the HandScan (professionals working at the developing company Demcon Hemics) on the technical properties and functionalities. A patient with RA critically examined a draft version of the survey and improvements were made based on the feedback.

The survey was constructed with the survey software Qualtrics XM (Qualtrics, Provo, UT). Patients could fill out the survey online, download a version to print out, or have a paper version sent to their home upon request. The survey consisted of 31 closed and open-ended questions, covering three different topics and displayed on 9 pages while participants could track their progress. Patients could change their answers and use a button to go back to previous answers.

- Topic 1 concerned the current monitoring process, ie the process of monitoring disease status and inflammatory activity.
- Topic 2 concerned the perceived added value of the HandScan.
- Topic 3 concerned the patient’s perspective on active involvement of patients in future research for the development of technology. The results of this topic will serve as a first investigation for a future project. As the topics covered in part 3 are out of the scope of the current study, results will not be presented in this paper.

For the complete survey (translated into English) see Supplementary material 1.

### Data analyses

Only completed surveys were used for analysis.

A person who underwent at least one HandScan test and discussed the results with a rheumatologist was considered as having experience with the HandScan.

Free-text comments as answers to open-ended questions were categorized. For this goal the first and second authors familiarized themselves with the free-text comments and identified suitable categories. Thereafter they independently assigned 10% of the free-text comments into the created categories. This resulted in an agreement of 92%. Resulting differences were discussed until consensus was reached. The remaining free-text comments were categorized by the first author. Furthermore, for relevant questions, free-text comments were categorized as being ‘positive’ or ‘negative’.

Data were analysed using descriptive statistics in SPSS (version 26). Comparisons of the perceived added value of the HandScan between patients with and without experience with the HandScan were analysed using a Chi-square test.

## Results

### Demographics

The completion rate of the survey was 64%, with a large part of drop-outs due to patients who only agreed to participate but not answer any questions. The survey was completed by 408 respondents, on a mobile device (*n* = 279), a desktop (*n* = 122) or paper (*n* = 7). Three-hundred and sixty-two respondents had no previous experience with the HandScan (89%), whereas 46 respondents (11%) did. Further characteristics of the respondents of the survey are depicted in Table 1.

**Table 1:** Description of the 408 respondents

Characteristic	Mean (SD)/*N* (%)
Age	54 (13.7)
Gender	
Women	354 (87%)
Men	54 (13%)
Years since RA diagnosis	12 (11.0)
Self-reported severity of RA, scale 0–10	5.3 (2.4)
Main complaints	
Mainly joints in hand and wrist	90 (22%)
Mainly other joints	31 (8%)
Both hand and wrist and other joints	280 (69%)
No complaints in joints	7 (2%)
Education level	
No or primary education	14 (3%)
Secondary education	91 (22%)
Intermediate education	153 (38%)
Higher education	148 (36%)
No response	2 (1%)
Technology adoption type	
Innovator	11 (3%)
Early adopter	147 (36%)
Early majority	177 (43%)
Late majority	49 (12%)
Laggards	23 (6%)
No response	1 (0%)

Technology adoption type based on the diffusion of innovation theory [17]

### Current monitoring process

#### Visits to outpatient clinics

Most respondents visited the outpatient clinics 1–4 times per year [1–2 times *n* = 171 (42%); 3–4 times *n* = 164 (40%)], and 372 respondents (91%) were satisfied with the number of visits. The most frequently visited healthcare professional was a rheumatologist, followed by a rheumatology nurse.

#### Examinations during visits

Respondents reported that during their visits physicians almost always (96%) asked for a patient’s opinion on the current disease status, and that blood testing was in general conducted (90%). A physical examination of the joints occurred almost always according to 71% of respondents. In general, respondents were satisfied (86%) with the frequency of being asked their opinion, blood tests, and physical examinations. Imaging (eg radiography, ultrasound, CT, MRI) occurred either on some visits (42%) or almost never (48%). Two-hundred and eighteen respondents (59%) were satisfied with the frequency of imaging.

Based on the percentage of respondents that answered ‘agree’ or ‘completely agree’, respondents indicated that the healthcare professional discussed the outcomes of the examinations during hospital visits with them (*n* = 360, 88%), that they understood the results of the examinations (*n* = 359, 88%), and also understood what these results could mean for their treatment (*n* = 334, 82%). Furthermore, 345 respondents (85%) indicated that healthcare professionals took sufficient time to answer their questions. Three-hundred and thirty (81%) respondents trusted the outcomes of the tests to inform decisions on treatment. Respondents who were already examined using the HandScan had more trust in the results of the HandScan for decisions on their treatment, *χ*^*2*^ (5, *n* = 408) = 44.6, *p* < 0.001* than respondents without this experience.

#### Insight in the status of the disease

Out of the total of 408 respondents, 298 (73%) believed they had sufficient insight into the current status of their RA. Respondents who found the frequency of imaging too low are less likely to feel they have sufficient insight into their RA activity, *χ*^2^ (4, *n* = 367) = 47.4, *p* < 0.001* compared with respondents satisfied with this frequency.

One hundred and two respondents (28%) would like to receive additional information from their rheumatologist. The respondents who felt to have insufficient insight were more likely to report to desire additional information (27 out of 43, 63%) compared to those who felt to have sufficient insight (50 out of 261, 19%, *χ*2 (1, *n* = 304) = 37.2, *p* < 0.001*). Respondents indicated in the free-text comments that they would like to receive additional information on their current disease status (*n* = 29, 26%; including information on disease activity, the meaning of test results, or the choice of treatment). A further 26 respondents (23%) would like to receive (more) information on additional or alternative treatment, including information on the effect of nutrition or exercise on disease activity, or the possibilities of using medication with less side effects. A third category included information on side effects of medication and on extra-articular complaints of RA, such as pain and fatigue (*n* = 13, 12%). Other areas included long-term expectations for symptoms (*n* = 8, 7%), insight into progress of the disease (*n* = 6, 5%) and a general desire to have more imaging (*n* = 6, 5%).

#### Shared decision-making process

Respondents gave the shared decision-making process on average an 8.1 on a scale from 0–10 (median score 8; IQR 7–9). Only 24 respondents (6%) gave a score of 5 or lower, which could be considered as ‘unsatisfactory’. Of all free-text comments (304 respondents with in total 342 comments) regarding shared decision-making, 267 (78%) could be categorized as positive, 53 (15%) as negative and 22 (6%) were unclear or did not refer to the decision-making process. Respondents who gave the process a score of 5 or lower only provided free-text comments categorized as negative (*n* = 18), whereas respondents who gave the process a 6 or higher provided both positive (*n* = 267) and negative (*n* = 35) comments. Reasons to be satisfied included receiving sufficient explanations, good collaboration and communication with the rheumatologist (*n* = 214, 80%), satisfaction with the current status of their disease (*n* = 20, 7%), trust in the expertise of the rheumatologist (*n* = 16, 6%), adequate actions by the rheumatologist, personalized treatment (*n* = 10, 4%) and easy access to the rheumatologist (*n* = 7, 3%). Reasons to be dissatisfied included free-text comments on the collaboration and communication by the rheumatologist (*n* = 33, 62%), disagreeing with the rheumatologist’s decisions (*n* = 6, 11%), difficulties accessing the rheumatologist (*n* = 3, 6%), that the rheumatologist did not take all complaints into consideration (*n* = 3, 6%), not liking the treatment (*n* = 2, 4%), or other (*n* = 6, 11%).

### New imaging technology HandScan

#### Potential added value of the HandScan

When asked about the added value of the HandScan, 240 respondents (59%) reported a large added value, 148 (36%) a small added value, and 20 (5%) no added value. Respondents with experience with the HandScan were more likely to see a large added value than respondents without experience with the HandScan (*χ*^*2*^ (2, *n* = 408) = 6.7, *p* = 0.04*). Respondents who thought that the current frequency of imaging was too low, reported greater added value for the HandScan, *χ*^*2*^ (2, *n* = 367) = 11.5, *p* = 0.003* than those satisfied with this frequency. 345 respondents (85%), added 396 free-text comments (330 categorized as positive, 16 as negative comments). The negative comments included the following considerations: the HandScan is limited to the hand and wrist area (*n* = 8, 50%), the outcome would most likely not affect their treatment (*n* = 4, 25%), or other (*n* = 4, 25%). Positive comments included that the HandScan would provide a clear image of (the location of) inflammatory activity, i (*n* = 232, 70%). Other reasons why the HandScan could add value were the ability to contribute to early recognition of inflammation and adaptation of the treatment (*n* = 27, 8%), to show progression over time and perhaps predict future developments (*n* = 25, 8%), and other (*n* = 46, 14%).

#### Desired functionalities and possible improvements

In the free-text comments, respondents gave several suggestions for desired functionalities of the HandScan (respondents without experience with the HandScan; 310 free-text comments by 262 respondents) or possible improvements (respondents experienced with the HandScan; 22 free-text comments by 22 respondents). These can be found in Table 2.

**Table 2:** Desired functionalities and possible improvements of the HandScan

Desired functionalities (total of 310 free-text comments)	*N* (%)
Give a concise localization and a value for severity of the inflammation	109 (35%)
Visualize aspects of RA other than inflammation, such as damage to the joints, tendons, or connective tissue, to measure the temperature of the joints, excess fluids, bone density, blood flow, or loss of function (which are currently not features of the HandScan)	42 (14%)
Be used on every visit to monitor and accomplish early detection of inflammation	21 (7%)
Guide treatment and prevent further damage	16 (5%)
Give patients an explanation during the test, watch the screen during the test and/or get a printout for themselves and other physicians	14 (5%)
Other	56 (18%)
Unable to answer without gaining experience with the HandScan	52 (17%)

Possible improvements (total of 22 free-text comments)	*N* (%)
Do not know	12 (55%)
Use more frequent	7 (32%)
Other	3 (15%)

#### Preferred frequency of HandScan examinations

When asked on the preferred frequency of the use of the HandScan in the monitoring process, the most common answer was ‘at every hospital visit’ (*n* = 192, 47%), followed by ‘on some hospital visits’ (*n* = 171, 42%), ‘more often than just during hospital visits’ (*n* = 17, 4%), or ‘never’ (*n* = 9, 2%). Nineteen respondents (5%) answered that they had no opinion on this topic. Respondents who thought that the frequency of imaging was too low were more likely to report that they would prefer a HandScan at all check-ups, *χ*^*2*^ (4, *n* = 367) = 15.3, *p* = 0.004*. There was no difference between respondents with and without experience with the HandScan on the preferred frequency of a scan, *χ*^*2*^ (4, *n* = 408) = 2.1, *p* = 0.73.

#### Preference for HandScan examinations versus physical examinations

Out of the 408 respondents, 131 (32%) preferred the Hand-Scan, 123 (30%) had no preference, and 102 (25%) preferred a physical examination (and 52 respondents (13%) answered no opinion).). There was no difference between respondents with and without experience with the HandScan on whether they preferred the HandScan over a physical examination, *χ*^*2*^ (1, *n* = 233) = 1.7, *p* = 0.19. Those who preferred the HandScan provided free-text comments in support (130 comments in total), in which they considered the HandScan as more objective and accurate (*n* = 67, 52%), more convenient (less strenuous, less painful and faster; *n* = 43, 33%) and/or as a way of making inflammation visible for the respondents (*n* = 5, 4%), or useful since the respondents mostly had complaints in their hands/wrists (*n* = 5, 4%), and other (*n* = 10, 8%). In the 93 free-text comments on why respondents did not have a preference, they mentioned that if results were similar, both have pros and cons (*n* = 24, 26%), it depended on the complaints (*n* = 15, 16%), and they rather pointed out the complaints themselves instead of letting a device determine the symptoms (*n* = 5, 5%), and other (*n* = 7, 8%). Some respondents explained that no choice could be made without personally experiencing the HandScan (*n* = 12, 13%), and 30 respondents (32%) wrote that they would prefer to have both a physical examination and an examination with the HandScan. In 95 free-text comments respondents expressed why a physical examination was preferred. These included the following: that because other joints than the ones in their hands were of importance as well (*n* = 57, 60%), respondents had more trust in the rheumatologist than in a device (*n* = 13, 14%). Furthermore some respondents highlighted the importance of human contact during the physical examination (*n* = 4, 4%) and expressed their opinion that optical technology cannot observe everything (*n* = 4, 4%) and other (*n* = 17, 18%). Of note, across all categories, 40 respondents (10%) stated in the comments that they would prefer a combination of the HandScan and a physical examination, even though this answer option was not provided in the survey.

## Discussion

The survey results showed that patients were in general satisfied with the current monitoring process. It was suggested by patients that their insight into their disease could be improved and furthermore that the HandScan was a valuable addition to the monitoring process of rheumatoid arthritis in a secondary care setting.

### Current monitoring process

The results of the current survey showed that the majority of patients is satisfied with the number of medical appointments, physical examinations and blood tests. However, not all patients are satisfied with their perceived insight into the current status of their disease. They indicated a desire for additional information about the current status of their disease, what this status means for the choice of treatment, and possible (additional) treatments. These results are in line with previous studies among patients with RA that consistently show a high need for information [7, 18–20].

Insight into the current status and possible treatments is an important prerequisite for patient participation in treatment decisions [8, 21]. The level of involvement in decisions concerning their treatment was perceived as good by the patients in this survey. Although this is in agreement with previous research, it should also be noted that patients with RA are not always aware of the various levels of participation they potentially could have [8]. Therefore, high satisfaction with the current process does not necessarily mean there is no room for improvement. It is important to investigate methods to increase patients’ awareness on active involvement as well as improving their insights into their disease, as these are prerequisites for them to engage in shared decision-making with their healthcare professionals [4, 8].

According to the patients participating in this survey, increasing the frequency of imaging is one way to obtain more insight into the current status of their RA. More frequent imaging could provide patients with a better understanding of the current level and location of inflammation. A substantial group of patients would like to have more imaging and this was especially true for patients who believed they did not have sufficient knowledge on the current status of their RA. The use of imaging is also recommended by the EULAR for the clinical management of RA. These organizations state that imaging can be useful in disease monitoring, since inflammation as seen on imaging may be more predictive of therapeutic response than clinical features and it can predict future flare-ups of inflammation even when a patient seems to be in remission [10]. Thus, more frequent imaging may be used to better inform patients on the current status of their disease and to monitor effects of treatment over time.

### New imaging technology—HandScan

Imaging as part of the monitoring process in RA is mostly done with radiography, ultrasound, CT, MRI, or PET [10]. In general, patients believed that a device based on optical spectral transmission—the HandScan—can be a valuable part of the monitoring process.

Almost all patients saw either a small or large added value of the HandScan in their current monitoring process. This was mostly due to the ability of the HandScan to detect the presence or absence of inflammation for each joint separately and its ability to calculate a score for the severity of the inflammation. Several patients indicated that they would appreciate the opportunity to find out whether their suspicions regarding the cause of pain or the presence of inflammation in a specific joint could be verified or disproven. This type of information can increase the insight into the current situation and can also be used for treatment decisions.

Patients were split on their preference for HandScan examinations or physical examinations. The HandScan was considered more objective, accurate and convenient, whereas physical examinations would also include joints outside of the hands and include the expertise of the rheumatologist. These arguments may also explain why a substantial group of patients would prefer a combination of both examinations.

Recognizing inflammatory activity early and treating accordingly is important for a tight control of RA [4]. The HandScan might provide additional information to support such an approach, especially if frequently performed. The majority of patients preferred to make a scan either at every hospital visit or most hospital visits. Due to the objective nature of the measurements, results of the scan can be tracked over time to show the pattern and severity of inflammation. Further research among different stakeholders should determine whether and how integration of the HandScan into existing monitoring and treatment procedures is cost-effective.

One important caveat is that the HandScan only measures joints in the hands and wrists. These joints are commonly affected in RA, and in this study almost all patients reported that these joints were involved. Thus although the HandScan does not cover all joints, it will be relevant to a large proportion of the RA population.

### Limitations

Some limitations should be considered. First, due to measures surrounding the Covid-19 pandemic most of the recruitment now occurred online, which may have resulted in the exclusion of patients with limited digital literacy. However, data content was not affected as the survey focused on the period before implementation of these measures. Further, although asked about the situation pre-pandemic, we cannot exclude the possibility of patients answering with the situation during the pandemic in mind. Second, 87% of the respondents were women, which is higher than the 67% women among the general RA population [2]. This could be a result of our digital recruitment procedures, as women are more active on social media in the area of health [22]. However, subanalyses of the data in this study did not suggest a difference in opinion between men and women (data not shown). It is, therefore, unlikely that the overrepresentation of women in the current study strongly affected the study findings. Third, only a small percentage of the respondents based their answers on personal experience with the HandScan, whereas the majority based their responses on description alone. Forming an opinion based on theory is more difficult than based on experience. We found that patients with experience saw more added value of the HandScan and have higher trust in the outcomes of the HandScan compared to those without experience, but future research should be conducted among a larger group of patients who underwent a HandScan.

### Conclusion

Patients with RA are in general satisfied with the current monitoring and decision-making process. However, they believe their understanding of the current status of their disease could be improved and would like to increase the frequency of imaging. More frequent imaging might give patients with RA better insight into their disease activity, empowering them to take a more active role in the decision-making process. Patients believe that frequent imaging based on optical spectral transmission—such as with the HandScan—could be a valuable addition to their monitoring process, especially due to the possibilities to measure the presence and severity of inflammatory activity for each separate joint.

## Supplementary Information

Below is the link to the electronic supplementary material.

Supplementary file1 (DOCX 34 kb)

## Data Availability

The data of the survey will be published in the 4TU-repository under the https://doi.org/10.4121/14877066.
