# Supplementary material for: Monitoring of rheumatoid arthritis: a patient survey on disease insight and possible added value of an innovative inflammation monitoring device
Source: Rheumatol Int. 2021 Oct 21;42(9):1565–72. doi: 10.1007/s00296-021-05026-8 (PMC8530199; doi:10.1007/s00296-021-05026-8)
Supplement: Supplementary file 1 — Supplementary file1 (DOCX 34 kb) [file 296_2021_5026_MOESM1_ESM.docx]

## Supplementary material 1: Survey rheumatoid arthritis and the HandScan (translated from Dutch to English)

1. How long have you been diagnosed with rheumatoid arthritis?

2. On a scale of 0 to 10, what was the severity of rheumatoid arthritis symptoms in the past month?

3. Which joints give you symptoms?

O Mainly joints in the hand and/or wrist

O Mainly other joints

O Both joints in the hand and/or wrist

O I don’t have symptoms in my joints

4. Has any appointment for your rheumatoid arthritis checkup been changed due to the corona measures? (multiple answers possible)

O one or more appointments have been cancelled or rescheduled

O one or more appointments have been changed to a digital appointment or appointment by phone

O one or more appointments took place as scheduled

O no appointment had been scheduled since the start of the measures

5. In the year before the corona crisis, how often did you have an appointment with a health care provider at the hospital to check on your rheumatoid arthritis and inflammatory activity?

O never

O 1-2 times

O 3-4 times

O 5-6 times

O more often, namely…

6. How do you feel about the number of appointments?

O too many O good O too few

7. Which health care providers did you see during your appointments in the year before the corona crisis for monitoring your rheumatoid arthritis?

|  | How often? | | | | | I feel this is: | | |
| --- | --- | --- | --- | --- | --- | --- | --- | --- |
|  | Never | 1-2x | 3-4x | 5-6x | More often | Too often | Good | Too few |
| Rheumatologist | O | O | O | O | O | O | O | O |
| Rheumatology nurse | O | O | O | O | O | O | O | O |
| Nurse | O | O | O | O | O | O | O | O |
| Physician assistant | O | O | O | O | O | O | O | O |
| General practitioner | O | O | O | O | O | O | O | O |
| Other, namely… | O | O | O | O | O | O | O | O |

8. What tests were conducted during your appointments in the year before the corona crisis?

|  | How often? | | | I feel this is: | | |  |
| --- | --- | --- | --- | --- | --- | --- | --- |
|  | (almost) always | sometimes | (almost) never | Too often | Good | Too few | |
| Blood tests | O | O | O | O | O | O | |
| Physical examination of joints | O | O | O | O | O | O | |
| Imaging (e.g., ultrasound, CT scan, MRI or HandScan) | O | O | O | O | O | O | |
| The doctor asked you about how it is going | O | O | O | O | O | O | |
| Other, namely… | O | O | O | O | O | O | |

9. Can you indicate the extent to which you agree with the statements below?

|  | Strongly disagree | Disagree | Neutral | Agree | Totally agree |
| --- | --- | --- | --- | --- | --- |
| The health care provider discusses the results of the examination with me | O | O | O | O | O |
| I understand the results of the examination | O | O | O | O | O |
| I understand what the outcomes mean for the treatment of my rheumatoid arthritis | O | O | O | O | O |
| I have enough time to ask all my questions during the appointment | O | O | O | O | O |
| I have sufficient understanding of how active my rheumatoid arthritis is | O | O | O | O | O |
| I trust the results of the studies enough to take them into account in the decisions surrounding my treatment | O | O | O | O | O |

10. Would you like additional information from the health care professional?

O no O yes, namely…

11. On a scale of 0 to 10, how satisfied are you with the extent to which you are involved in decisions about your treatment?

12. Why are you satisfied or dissatisfied?

*The HandScan*

*The HandScan is a new device developed by Demcon Hemics. The HandScan measures the blood flow in the joints of the hand and wrist. The blood flow is a measure of inflammation and thus indicates how active the rheumatoid arthritis is at the moment. The measurement takes place in the hospital where the HandScan is operated by a healthcare professional. You place your hand in the device, after which a completely harmless light beam measures the blood flow to your joints. The blood flow to the hand is slightly restricted (similar to a blood pressure device). The examination takes less than 5 minutes and is painless. The HandScan objectively indicates with a number and an image how active the rheumatoid arthritis is in the entire hand and per joint.*

13. Has your inflammatory activity been examined by the HandScan before?

O no

O yes, at my usual checkups

O yes, but only as part of a scientific study

1. If yes, how often?
2. If yes, what experiences do you have with the HandScan?

O a scan has been made of my hand and wrist

O a scan has been made and the results were discussed with me

O a scan has been made, the results were discussed and the results have been taken into account in the decision about follow-up treatments

14. Do you think that the HandScan can be of added value in monitoring your inflammatory activity?

O no O yes, a small added value O yes, a large added value

1. Why?

15. What could the HandScan add?

16. What function should the HandScan certainly have?/How can the HandScan be improved?*

**question dependent on experience with HandScan (Q13)*

17. Would you like the HandScan to be used at checkup appointments?

O no

O yes, but it is not necessary to do this at every checkup appointment

O yes, preferably at every checkup appointment

O yes, preferably more often than only at check-ups

O no opinion

18. If the results are the same, would you prefer a physical examination (where the doctor checks all joints) or a measurement with the HandScan?

O physical examination, because…

O HandScan, because…

O no preference, because…

O no opinion

19. Do you trust the results of the HandScan enough to take them into account in a decision around your treatment?

0 Strongly disagree 0 Disagree 0 Neutral 0 Agree 0 Totally agree

*The best use of the HandScan in the care of people with rheumatoid arthritis is currently being investigated. The results will be used to determine how the HandScan can be adapted or further developed. The following questions are about your willingness to participate in any follow-up research. By answering these questions, you are not making any commitment.*

20. To what extent do you agree with the following statements?

|  | Strongly disagree | Disagree | Neutral | Agree | Totally agree |
| --- | --- | --- | --- | --- | --- |
| I think it is important for patients to be involved in research on new technology within the care of rheumatoid arthritis | O | O | O | O | O |
| I think it is important for patients to be involved in research on the HandScan | O | O | O | O | O |
| I find it important that I personally can be involved in research on the HandScan | O | O | O | O | O |

21. Would you be willing to participate in research on the HandScan?

O no O yes, once O yes, more often

22. What are reasons for you wanting or not wanting to participate in research on the HandScan?

23. What conditions are important to you in deciding whether or not to participate in research on the HandScan? *(Consider compensation, travel expenses, ability to participate from home, the role you would have, etc.)*

24. What is your gender?

O male O female O other

25. What is your age?

26. In which province do you live?

O Groningen O Drenthe O Utrecht O Zeeland O Friesland O Flevoland O Noord-Holland O Noord-Brabant O Overijssel O Gelderland O South Holland O Limburg

1. If Overijssel, do you live in Twente?

O yes O no

27. What is your highest completed education?

O no education

O elementary school, primary education

O secondary school

O intermediate education

O higher education

O other, namely…

28. Below are statements about the use of technology. Choose the statement that suits you best.

O I am usually ahead of the rest and often have innovative ideas. I am always the first to use a new technology.

O I like to try out new things and I like to experiment with new technology

O I only use a new technology when I know it is convenient and useful. I will then also recommend it to others

O I will only use a new technology if many people also use it and I see that it is convenient and useful to use

O I do not use a new technology quickly because I like to stick with what I already know and use

29. Do you have any questions or comments? For example, are there any questions you missed?

30. Where did you find this questionnaire?

O my health care professional pointed it out to me

O via a flyer

O via social media

O via an acquaintance

O via the website of ReumaNL

O via the patients' association

31. May we contact you in the future for any of the options listed below?

O Yes, I would like to receive a summary of the survey

O Yes, I am okay with being contacted for possible follow-up research. (We will send you information about new research in the future. You can decide at that time whether or not you want to participate in that research).

O Yes, I would like to have a chance to win a gift card of 10 Euros and you may contact me in case I won.

O No

1. Please fill out your email address:

*Thank you for your participation in this survey.*
